# Cystic hygroma and anaesthetic implication

**DOI:** 10.4103/0019-5049.76568

**Published:** 2011

**Authors:** Geeta Kamal, Aikta Gupta, Sapna Bathla, Neelam Prasad

**Affiliations:** Department of Anaesthesiology, Chacha Nehru Bal Chikitsalaya (Affiliated to Maulana Azad Medical College), Delhi, India

Sir,

Cystic hygroma is a benign tumor composed of large lymph containing cysts.[1] Lymphanigomas of head and neck region frequently present challenges to the anaesthesiologists due to extension in the neck, airway and thorax. We describe the difficulties encountered in intubation and postoperative care of the patient.

**Figure 1:**
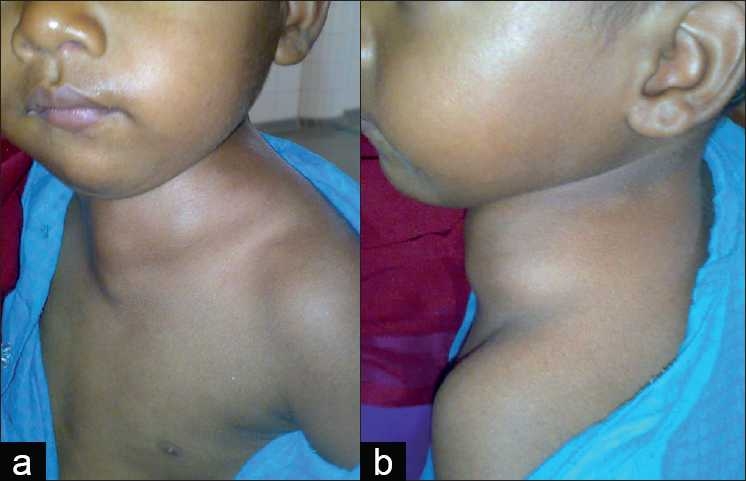
(a) Front and (b) side view of the patient showing extent of cystic hygroma

A 2-year-old, 10kg child presented with a painful and progressively increasing cystic mass (4×2cm) on the left side of the neck extending beyond the midline [Figure 1a and b]. A diagnosis of cystic hygroma was made and surgical excision was planned after no response to intralesional bleomycin.

On examination patient had mild respiratory distress. Anteroposterior (AP) and lateral X-ray of neck showed minimal compression and deviation of trachea to right side. X-ray chest showed soft tissue mass in superior mediastinum. Informed consent for anaesthesia was taken and parents were explained about anticipated difficult intubation. After preoxygenation with 100% oxygen for 5 min, the patient was induced with injection atropine, fentanyl and propofol. There was no difficulty in mask ventilation. Intubation was tried first with number 4.5 uncuffed flexomettalic endotracheal tube (ETT) followed by 4, with one assistant lifting the mass from the trachea during the procedure. ETT could be passed beyond the vocal cords but not the subglottic region. As patient was maintaining saturation we planned to intubate using succinylcholine. After ventilating the patient with 100% oxygen we reattempted the intubation with the number 4 uncuffed flexomettalic tube requesting the assistant to lift and pull the mass to left side in proper manner. This time the tube could be easily passed beyond the subglottic region. The tube was fixed at 10 cm after confirming the air entry. Intraoperative period was uneventful and the cystic lesion extending upto tracheal wall was excised. At the end of surgery after reversal, as the patient was fulfilling extubation criteria, he was extubated and shifted to pediatric intensive care unit (PICU) for monitoring. In PICU, the child was stable for initial 36 hrs, but then suddenly became drowsy, respiratory rate increased to 50-60/minute and saturation dropped to 80-85%. Intensivists had a dilemma about the cause of patient’s deterioration which could be due to upper airway obstruction by laryngeal oedema or collapse of lower airway by intrathoracic cystic hygroma. Bedside emergency fibreoptic bronchoscopy revealed severe oropharnygeal, arytenoid and laryngeal oedema and collapse of bronchial wall on left side. Patient was reintubated and kept on mechanical ventilation in view of massive oedema. Although patient demonstrated signs of initial improvement, his condition deteriorated again and expired after 4 days.

As cervical cystic hygroma are always at risk of causing airway compromise, such patients are challenging to the anaesthesiologists. This case highlighted the anaesthetic implications of these patients undergoing surgery.

A key to proper management of such patients is direct communication between the surgeon, anaesthesiologists and parents. Educating the parents on their child’s potential presentation and complication of the mass is very important.

Advanced preoperative preparation including detailed examination (evaluation of congenital anomalies, signs of airway compromise and associated radiological evaluation) decreases morbidity.[2] Experienced anaesthetist as expert assistant is essential. Awake fibreoptic intubation is the technique of choice, but potentially difficult and traumatic in infants.[3] It is necessary to maintain spontaneous ventilation in the presence of airway infiltration and compression at least until the airway has been secured. Inhalation anaesthesia in an acceptable alternative incase of difficult intubation. Stress-induced physiological changes such as increase in heart rate, blood pressure, oxygen consumption are always a concern.[4] Succinylcholine was used to facilitate the intubation only after confirmation that patient could be mask ventilated. Postoperative ventilation may be necessary where there is injury to the larynx or the recurrent laryngeal nerve, or excision of large cysts. Anaesthetists should contemplate elective postoperative ventilation after excision of large lesions until airway oedema has subsided or where residual lesion may be anticipated. We have extubated our patient but elective ventilation could have altered the prognosis.

In conclusion, successful results require proper preoperative evaluation and intraoperative management along with assistance of experienced anaesthetist to avoid the morbidity and mortality.
